# Modified moving average methodology applied to the treadmill stress testing analysis of microvolt T-wave alternans

**DOI:** 10.1038/s41598-022-26535-x

**Published:** 2022-12-27

**Authors:** Horacio Gomes Pereira Filho, Nelson Samesima, Bruna Affonso Madaloso, Nancy Maria Martins de Oliveira Tobias, Mirella Facin, Carlos Alberto Pastore

**Affiliations:** Instituto do Coracao (InCor), Hospital das Clinicas HCFMUSP, Faculdade de Medicina, Universidade de Sao Paulo, Unidade de Eletrocardiografia, Sao Paulo, SP 05403-900 Brazil

**Keywords:** Cardiovascular biology, Cardiovascular diseases, Arrhythmias, Ventricular fibrillation, Cardiology, Medical research, Biomarkers

## Abstract

Sudden cardiac death is impactful. There has been an increase in the search for tools capable of identifying individuals who are most susceptible, such as the microvolt T-wave alternans. This study aims to analyze the applicability of the modified moving average methodology to obtain the microvolt T-wave alternans using treadmill specific protocols. Medical records of patients during the period August 2006–December 2014 were retrospectively analyzed. Five hundred and thirty nine exams were then included, divided into groups according to the protocol and updating factor used: Ellestad factor 8 or 32, Naughton factor 8 or 32. The topics for analysis were the alternans behavior, noise and confirmation according to the groups of leads analyzed (frontal, transversal and orthogonal planes). The greater microvolt T-wave alternans was found during the stress phase in most of the tests. Group Naughton 8 presented lower noise in this phase for the transverse and orthogonal planes (p = 0.0082 and p < 0.0001), with greater confirmation of frontal and orthogonal planes in comparison with group Ellestad 8 (p = 0.0002 and 0.0008). The results indicate the viability of simultaneous performance of the stress test and measurement of the T wave alternans with Naughton protocol with 1/8 updating factor.

## Introduction

Sudden cardiac death (SCD) currently represents a great challenge to Medicine. Its occurrence in apparently healthy individuals and sometimes sportsmen has an immeasurable impact due to the physical, emotional and social damage caused to the patient, their family and society in general. The occurrence is estimated at about 5 million events per year, with an incidence of 50 to 100 cases per year per 100,000 people^1,2^. Among the most common causes of SCD are the manifestations of coronary heart disease, dilated cardiomyopathy, hypertrophic cardiomyopathy, valvular diseases, channelopathies and other genetic diseases. On many occasions, results of initial investigation or screening tests, such as the resting electrocardiogram, are absolutely normal.

This fact has led to a search for early detection mechanisms to recognize individuals with potential risk for SCD, and new modalities of exams and procedures have been considered among the tools of cardiovascular investigation, such as the analysis of microvolt T-wave alternans (TWA)^3^ in the last years. TWA consists in the analysis, through specific software, of the beat-to-beat changes of the ST-segment and T-wave that are not detected to the naked eye^4–9^.TWA accesses indirectly the increased dispersion of action potentials in the heart cells, which are the basis for the reentry and ventricular fibrillation mechanisms.

Among the TWA analysis systems, the Modified Moving Average (MMA) methodology described in 2002 by Verrier and Nearing has been used in different forms in the clinical practice, such as by exercise test and 24-h Holter, with some interesting characteristics: more accessible, cheap, easy comprehension and allow the examiner to confirm its results, in opposition to the spectral method^10,11^. About the use of MMA methodology for the assessment of TWA there is a large reference trial, FINCAVAS, that since 2007^12^ has analyzed overall, by 2011, almost 3600 individuals using cycle ergometer. However, there were some questions about methodological information, such as the influence of noise in the results and how the TWA measurement was confirmed, that were not described. In many facilities around the world the treadmill is the most common ergometer, and some variables such as “exercise capacity” and “maximum heart rate” have higher measurements than those with a bicycle ergometer. Besides, there are few studies about the use of MMA in TWA and conventional exercise protocols of ergometry.

TWA monitoring has been evaluated over the years for both techniques. For the MMA, when applied for the 24-h Holter methodology, it is recommended the recording and analysis of the V precordial leads, and for the bicycle exercise test, the evaluation of the transverse plane, especially the V5 lead^3,5^. The spectral method measures TWA using precordial V leads and the 3 orthogonal leads X, Y, Z^8,10^. However, the exercise stress test commonly evaluates the electrocardiogram in the frontal and transverse plane leads, and may include more leads, such as Frank's orthogonal leads.

On the other hand, the possibility to combine the advantages of the treadmill exercise test (low cost, easy acquisition and reproducibility) and the measure of TWA represents an important and very practical instrument for assessing the risk of SCD.

## Objective

To analyze the applicability, technical feasibility and methodological aspects of the MMA method to obtain microvolt T-wave alternans (TWA) through specific protocols in treadmill exercise test and with different updating factors. Therefore, we evaluated whether obtaining the TWA by the MMA method in an ergometry routine would be feasible, comparing an intense protocol and another attenuated one, with different updating factors (high or low sensitivity), to verify the influence of treadmill exercise in the measurement of TWA considering these parameters.

## Results

**Figure 1:**
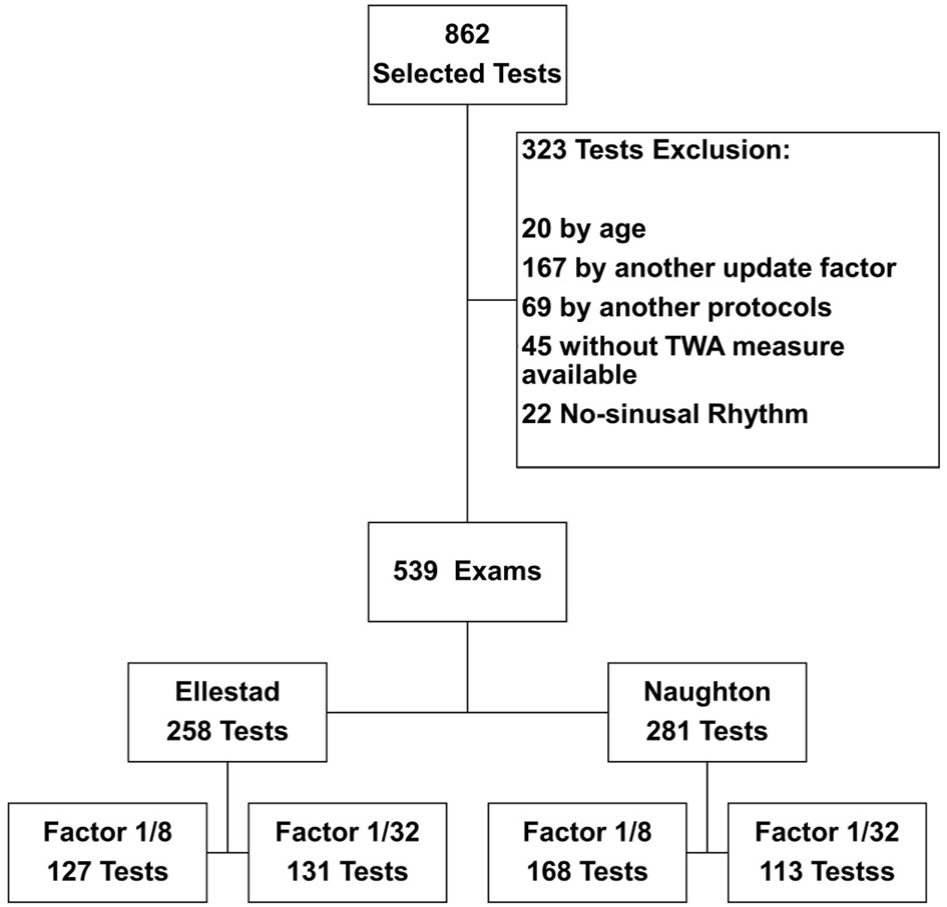
Study flowchart and distribution of the number of tests per group.

Initially, 862 exams were selected, of which 323 (37.4%) were excluded: 20 by age, 167 performed with another update factor, 69 performed in other exercise protocols, 45 without the TWA record available, and 22 by a non-sinus rhythm at pre-test ECG. Thus, 539 exams were submitted to analysis, according to the flowchart below (Fig. 1).

The number of tests performed in Ellestad protocol was 258 (127 performed in factor 1/8 and 131 in factor 1/32), and for Naughton protocol a total of 281 exams (168 performed in factor 1/8 and 113 in factor 1/32) were performed.

### General table

Table 1 shows some characteristics of the studied sample:

**Table 1:** General characteristics of the studied sample.

Variable	Description (n = 539)
Men (%)	66.2
Age (years)	40.5 ± 14.1
Weight (kg)	73.0 ± 14.2
Height (cm)	168.5 ± 9.8
BMI (kg/m^2^)	25.7 ± 4.3
Exercise duration (s)	616.6 ± 211
MET	9 ± 3.8
Double product (bpmxmmHg/100)	254.4 ± 78
Resting HR (bpm)	73.9 ± 15
Maximum exercise HR (bpm)	144 ± 31.7
Maximal TWA HR (bpm)	104.9 ± 18.5

Values expressed in percentages (%) for male distribution, and mean followed by standard deviation for the other variables

*BMI* Body Mass Index, *HR* Heart Rate, *MET* Metabolic Equivalent of the Task (MET = VO2máx/3,5 − 1 MET = 1 kcal/kg/h).

### Occurrence during the test of maximum TWA

Table 2 shows the moment of the exercise test in which the highest TWA was recorded: rest, exercise or recovery, among the 4 groups.

**Table 2:** Distribution in percentage of the moment of occurrence of maximal TWA during the exercise test.

	Group E8 (n = 127)	Group E32 (n = 131)	Group N8 (n = 168)	Group N32 (n = 113)	Total (n = 539)	p*
**Maximal TWA (%)**						0.02
Rest	0/127 (0)	2/131 (1.5)	0/168 (0)	0/113 (0)	2/539 (0.4)	
Exercise	104/127 (81.9)	113/131 (86.3)	152/168 (90.5)	106/113 (93.8)	475/539 (88.1)	
Recovery	23/127 (18.1)	16/131 (12.2)	16/168 (9.5)	7/113 (6.2)	62/539 (11.5)	

Groups: E8 = Ellestad factor 1/8; E32 = Ellestad factor 1/32; N8 = Naughton factor 1/8; N32 = Naughton factor 1/32.

*Fisher’s exact test.

The majority of exams, 475 tests (88.1%), present the maximum TWA during the exercise phase, in greater proportion in groups N8 and N32 than E8 and E32 (90.5% and 93.8% against 81.9% and 86.3%, respectively). E8 group had the register of maximum TWA during recovery phase in 18.1% of the tests.

### Resting phase

Table 3 summarizes the aspects of the noise value and confirmation according to the set of leads (frontal, transverse and orthogonal leads) in the different groups.

**Table 3:** Noise values and confirmation during the resting phase, according to set of leads in the different groups.

Noise (µV)	E8 group n = 127	E32 group n = 131	p
Frontal leads	3.2 ± 2.7	4.2 ± 12.7	** < 0.0001**
Transverse leads	3.3 ± 8.2	1.8 ± 6.1	** < 0.0001**
Orthogonal leads	3.3 ± 2.3	2.2 ± 6.6	** < 0.0001**

Confirmation (%)			p
Frontal leads	126/127 (99.2%)	123/131 (93.9%)	0.04
Transverse leads	126/127 (99.2%)	124/131 (94.7%)	0.07
Orthogonal leads	125/127 (98.4%)	124/131 (94.7%)	0.17

Noise (µV)	E8 group n = 127	N8 group n = 168	p
Frontal leads	3.2 ± 2.7	4.4 ± 8.1	0.18
Transverse leads	3.3 ± 8.2	3.3 ± 8.5	0.57
Orthogonal leads	3.3 ± 2.3	3.1 ± 3.0	0.49

Confirmation (%)			p
Frontal leads	126/127 (99.2%)	167/168 (99.4%)	1.00
Transverse leads	126/127 (99.2%)	168/168 (100.0%)	0.43
Orthogonal leads	125/127 (98.4%)	165/168 (98.2%)	1.00

Noise (µV)	E32 group n = 127	N32 group n = 113	p
Frontal leads	4.2 ± 12.7	3.2 ± 8.3	0.72
Transverse leads	1.8 ± 6.1	1.7 ± 4.1	0.52
Orthogonal leads	2.2 ± 6.6	1.5 ± 1.2	0.68

Confirmation (%)			p
Frontal leads	123/131 (93.9%)	106/113 (93.8%)	1.00
Transverse leads	124/131 (94.7%)	107/113 (94.7%)	1.00
Orthogonal leads	124/131 (94.7%)	107/113 (94.7%)	1.00

Noise (µV)	N8 group n = 168	N32 group n = 113	p
Frontal leads	4.4 ± 8.1	3.2 ± 8.3	** < 0.0001**
Transverse leads	3.3 ± 8.5	1.7 ± 4.1	** < 0.0001**
Orthogonal leads	3.1 ± 3.0	1.5 ± 1.2	** < 0.0001**

Confirmation (%)			p
Frontal leads	167/168 (99.4%)	106/113 (93.8%)	**0.008**
Transverse leads	168/168 (100.0%)	107/113 (94.7%)	**0.004**
Orthogonal leads	165/168 (98.2%)	107/113 (94.7%)	0.16

Noise values are expressed as mean and standard deviation.

Groups: E8 = Ellestad factor 1/8; E32 = Ellestad factor 1/32; N8 = Naughton factor 1/8; N32 = Naughton factor 1/32.

Data presented as Mean ± Standard Error. Bold values indicate statistical significance.

Significant p-value considered 0.0083 (Bonferroni’s correction).

During rest phase it was observed that noise was significantly higher in groups with factor 1/8 (E8 and N8) in comparison with groups with factor 1/32 (E32 and N32) with p < 0.0001. Confirmation at this moment did not show differences between groups, with the exception of the comparison N8 vs N32 in which the latter had higher rates of invalidation in the frontal plane (p = 0.0008) and transverse (p = 0.0039).

### Maximum TWA moment

Table 4 summarizes the aspects of noise, confirmation and TWA values during this moment, by set of leads in each studied group.

**Table 4:** Noise values and confirmation during the maximum TWA moment, according to set of leads in the different groups.

Noise (µV)	E8 group n = 127	E32 group n = 131	p
Frontal leads	28 ± 31.9	44.1 ± 37.9	0.04
Transverse leads	16 ± 18.3	16.3 ± 22.4	0.01
Orthogonal leads	21.8 ± 40.6	14.4 ± 19.5	** < 0.0001**

Confirmation (%)			p
Frontal leads	84/127 (66.1%)	84/131 (64.1%)	0.80
Transverse leads	112/127 (88.2%)	125/131 (95.4%)	0.04
Orthogonal leads	92/127 (72.4%)	123/131 (93.9%)	** < 0.0001**

Noise(µV)	E8 group n = 127	N8 group n = 168	p
Frontal leads	28 ± 31.9	21.5 ± 58.9	0.04
Transverse leads	16 ± 18.3	12.7 ± 13.5	0.19
Orthogonal leads	21.8 ± 40.6	13.6 ± 18.9	0.01

Confirmation (%)			p
Frontal leads	84/127 (66.1%)	142/168 (84.5%)	**0.0002**
Transverse leads	112/127 (88.2%)	159/168 (94.6%)	0.05
Orthogonal leads	92/127 (72.4%)	148/168 (88.1%)	** < 0.0001**

Noise (µV)	E32 group n = 127	N32 group n = 113	p
Frontal leads	44.1 ± 37.9	23.8 ± 30.9	** < 0.0001**
Transverse leads	16.3 ± 22.4	11.3 ± 17.5	0.07
Orthogonal leads	14.4 ± 19.5	11.3 ± 15.6	0.97

Confirmation (%)			p
Frontal leads	84/131 (64.1%)	93/113 (82.3%)	**0.0002**
Transverse leads	125/131 (95.4%)	108/113 (95.6%)	1.00
Orthogonal leads	123/131 (93.9%)	105/113 (92.9%)	0.80

Noise (µV)	N8 group n = 168	N32 group n = 113	p
Frontal leads	21.5 ± 58.9	23.8 ± 30.9	0.076
Transverse leads	12.7 ± 13.5	11.3 ± 17.5	** < 0.0001**
Orthogonal leads	13.6 ± 18.9	11.3 ± 15.6	** < 0.0001**

Confirmation (%)			p
Frontal leads	142/168 (84.5%)	93/113 (82.3%)	0.63
transverse leads	159/168 (94.6%)	108/113 (95.6%)	0.79
orthogonal leads	148/168 (88.1%)	105/113 (92.9%)	0.23

Noise values are expressed as mean and standard deviation.

Groups: E8 = Ellestad factor 1/8; E32 = Ellestad factor 1/32; N8 = Naughton factor 1/8; N32 = Naughton factor 1/32.

Data presented as Mean ± Standard Error. Bold values indicate statistical significance.

Significant p-value considered 0.0083 (Bonferroni’s correction).

In this moment the noise in E32 group presented a higher average when compared to the N32 group in the isolated analysis of the set of frontal leads alone (p < 0.0001). Regarding the transverse plane, the groups with factor 1/8 (E8, N8) presented greater value noise than the groups E32 and N32 (p = 0.0082 and p < 0.0001, respectively), with the same aspects occurring in the orthogonal set leads. When the confirmation was analyzed in this moment the groups Naughton protocol (N8 and N32) presented greater frontal lead confirmation (84.5% and 82.3%) than Ellestad protocol groups E8 and E32 (66.1% and 64%), with p = 0.0002 and 0.0016 respectively. There were not statistical differences between the groups for the transverse set leads and E8 group that showed a lower confirmation rate when compared with the E32 (p < 0.0001) and N8 (p = 0.0008) for orthogonal set leads.

### Recovery phase

Table 5 summarizes the aspects of noise values and confirmation during this moment, by set of leads in each studied group.

**Table 5:** Noise values and confirmation during the recovery phase, according to set of leads in the different groups.

Noise (µV)	E8 group n = 127	E32 group n = 131	p
Frontal leads	30.4 ± 47.6	38.4 ± 38	0.32
Transverse leads	17.2 ± 29.8	21.2 ± 28.4	0.59
Orthogonal leads	16.1 ± 24	21.8 ± 29	0.05

Confirmation (%)			p
Frontal leads	74/127 (58.3%)	76/131 (58%)	1.00
Transverse leads	90/127 (70.9%)	95/131 (72.5%)	0.78
Orthogonal leads	88/127 (69.3%)	92/131 (70.2%)	0.89

Noise (µV)	E8 group n = 127	N8 group n = 168	p
Frontal leads	30.4 ± 47.6	11.7 ± 27.3	** < 0.0001**
Transverse leads	17.2 ± 29.8	7.1 ± 13.0	** < 0.0001**
Orthogonal leads	16.1 ± 24	9.1 ± 28.8	** < 0.0001**

Confirmation (%)			p
Frontal leads	74/127 (58.3%)	146/168 (86.9%)	** < 0.0001**
Transverse leads	90/127 (70.9%)	149/168 (88.7%)	**0.0002**
Orthogonal leads	88/127 (69.3%)	148/168 (88.1%)	** < 0.0001**

Noise (µV)	E32 group n = 127	N32 group n = 113	p
Frontal leads	38.4 ± 38	16.4 ± 26.8	** < 0.0001**
Transverse leads	21.2 ± 28.4	10.7 ± 21.6	** < 0.0001**
Orthogonal leads	21.8 ± 29	9.8 ± 22.8	0.97

Confirmation (%)			p
Frontal leads	76/131 (58%)	90/113 (79.6%)	**0.0003**
Transverse leads	95/131 (72.5%)	92/113 (81.4%)	0.13
Orthogonal leads	92/131 (70.2%)	92/113 (81.4%)	0.053

Noise (µV)	N8 group n = 168	N32 group n = 113	p
Frontal leads	11.7 ± 27.3	16.4 ± 26.8	0.08
Transverse leads	7.1 ± 13.0	10.7 ± 21.6	**0.007**
Orthogonal leads	9.1 ± 28.8	9.8 ± 22.8	** < 0.0001**

Confirmation (%)			p
Frontal leads	146/168 (86.9%)	90/113 (79.6%)	0.14
Transverse leads	149/168 (88.7%)	92/113 (81.4%)	0.12
Orthogonal leads	148/168 (88.1%)	92/113 (81.4%)	0.13

Noise values are expressed as mean and standard deviation.

Groups: E8 = Ellestad factor 1/8; E32 = Ellestad factor 1/32; N8 = Naughton factor 1/8; N32 = Naughton factor 1/32.

Data presented as Mean ± Standard Error. Bold values indicate statistical significance.

Significant p-value considered 0.0083 (Bonferroni’s correction).

The recovery phase was presented in 11.5% of the exams as the moment of highest registration of TWA. During this phase, the noise in frontal plane was significantly higher in the Ellestad groups when compared to Naughton groups. N8 group had a lower noise value in this set of leads (11.7 ± 27.3 microvolts), however it was not different than N32 group (p = 0.0833). In the isolate analysis of transverse plane set of leads, the Ellestad groups presented significantly higher noise values when compared to their counterparts in the update factor, in both comparisons (E8vsN8 and E32vsN32, p < 0.0001). N8 had the lowest average noise value in this set of leads and orthogonal set leads too when compared to the E8 and N32 groups (p < 0.0001).

When we analyzed the confirmation during the recovery phase, we observed that the set of frontal leads presented lower confirmation in groups E8 and E32 (58.3% and 58%, respectively) when compared with N8 and N32 (86.9% and 79.6%, respectively, with p < 0.0001 and p = 0.003). Regarding the transverse plane set of leads, there was a significant difference in the confirmation rate of the E8 (70.9%) and N8 (88.7%) with p = 0.0002. The same observation occurred in the analysis for orthogonal set leads, with a confirmation rate of 69.3% for the E8 group and 88.1% for the N8 group, with p < 0.0001.

### Maximum exercise phase

Table 6 provides the behavior of the variables analyzed during peak exercise.

**Table 6:** Noise values and confirmation during peak exercise, according to set of leads in the different groups.

Noise (µV)	E8 group n = 127	E32 group n = 131	p
Frontal leads	47.7 ± 40.4	89.9 ± 29	** < 0.0001**
Transverse Leads	31.1 ± 37.9	81.5 ± 45	** < 0.0001**
Orthogonal leads	32.2 ± 34.1	78 ± 32.3	** < 0.0001**

Confirmation (%)			p
Frontal leads	0/127 (0.0%)	1/131 (0.8%)	1.00
Transverse leads	0/127 (0.0%)	1/131 (0.8%)	1.00
Orthogonal leads	0/127 (0.0%)	3/131 (2.3%)	0.25

Noise (µV)	E8 group n = 127	N8 group n = 168	p
Frontal leads	47.7 ± 40.4	22.5 ± 36	** < 0.0001**
Transverse leads	31.1 ± 37.9	13.1 ± 17.3	** < 0.0001**
Orthogonal leads	32.2 ± 34.1	12.5 ± 15.2	** < 0.0001**

Confirmation (%)			p
Frontal leads	0/127 (0.0%)	67/168 (39.8%)	** < 0.0001**
Transverse leads	0/127 (0.0%)	75/168 (44.6%)	** < 0.0001**
Orthogonal leads	0/127 (0.0%)	73/168 (43.5%)	** < 0.0001**

Noise (µV)	E32 group n = 127	N32 group n = 113	p
Frontal leads	89.9 ± 29	47.3 ± 51.1	** < 0.0001**
Transverse leads	81.5 ± 45	27.4 ± 30.7	** < 0.0001**
Orthogonal leads	78 ± 32.3	27.2 ± 28.6	** < 0.0001**

Confirmation (%)			p
Frontal leads	1/131 (0.8%)	45/113 (39.8%)	** < 0.0001**
Transverse leads	1/131 (0.8%)	60/113 (53.1%)	** < 0.0001**
Orthogonal leads	3/131 (2.3%)	57/113 (50.4%)	** < 0.0001**

Noise (µV)	N8 group n = 168	N32 group n = 113	p
Frontal leads	22.5 ± 36	47.3 ± 51.1	**0.0001**
Transverse leads	13.1 ± 17.3	27.4 ± 30.7	0.09
Orthogonal leads	12.5 ± 15.2	27.2 ± 28.6	0.17

Confirmation (%)			p
Frontal leads	67/168 (39.8%)	45/113 (39.8%)	1.00
Transverse leads	75/168 (44.6%)	60/113 (53.1%)	0.18
Orthogonal leads	73/168 (43.5%)	57/113 (50.4%)	0.27

Noise values are expressed as mean and standard deviation.

Groups: E8 = Ellestad factor 1/8; E32 = Ellestad factor 1/32; N8 = Naughton factor 1/8; N32 = Naughton factor 1/32.

Data presented as Mean ± Standard Error. Bold values indicate statistical significance.

Significant p-value considered 0.0083 (Bonferroni’s correction).

During the moment of maximum exercise, the noise in the set of frontal leads presented its highest expression in the E32 group (89.9 ± 29 microvolts), while the lowest mean value was found in the N8 group (22.5 ± 36 microvolts), with p < 0.0001 in all comparisons performed. For the set of transverse leads, the highest noise value was again found in Ellestad groups, mainly E32 Group with an average of 81.5 ± 45 microvolts, but between the 2 Naughton groups there are no significant differences. The same aspect was found in the analysis of orthogonal leads, with lower values in the Naughton groups, but with no differences between N8 and N32.

As expected, when reaching higher heart rates, the Ellestad protocol did not allow any confirmation in E8 group for the 3 sets of leads during the maximal exercise phase, as well as the E32 group. The Naughtons groups did not differ among themselves in the different sets of leads, allowing confirmation of the TWA reading in about 39.9% and 39.8% of the tests in frontal set leads, 44.6% and 53.1% for the set of transverse leads and 43.5% and 50.4% of confirmation for orthogonal leads respectively for groups N8 and N32.

## Discussion

Exercise testing currently plays an important role in cardiovascular investigation, being widely disseminated in clinical practice, useful for the diagnosis of cardiovascular diseases, prognostic assessment, evaluation of symptoms and exercise-related arrhythmias, among others^13^. Its low cost and high reproducibility features make the exam universally accessible.

The TWA technique evaluates the existence of arrhythmogenic substrate, and is considered a clinical measure that assesses indirectly the action potential alternans of the cardiac myocardial cells. The action potential alternans is one of the factors contributing to the presence of the referred substrate^3,11^.

The initial clinical studies with TWA using the Spectral Method were with coronary artery disease and myocardial ischemia as risk factors for sudden cardiac death. These studies showed the high power of the negative predictive value of TWA^14–16^ and the important role in the non-invasive stratification of SCD^17–19^. FINCAVAS study using MMA showed a negative predictive value of 98.6% for sudden cardiac death^12^.

In the last years, the role of TWA use has substantially expanded to other investigation profiles, including the possibility of evaluating antiarrhythmic therapy^20^, risk stratification in genetic diseases such as Brugada Syndrome^21^, and Congenital Long QT^22^, hypertrophic cardiomyopathy^23,24^ and postoperative diseases^25^. The possibility of concomitant use of TWA techniques, during the performance of the exercise test showed to be very promising.

Particularly, the use of the MMA methodology in a cycle ergometer by FINCAVAS study^12,26^, by which the references values for TWA were established (e.g., TWA values above 47 mV were associated with higher global cardiovascular risk) pointed to a fast use of TWA measurement in the exercise test routine.

However, this same and important study did not aim to answer the methodological questions of performing the exam, measuring and confirming the results and reproducibility. In many parts around the world, the cardiovascular stress most performed is the treadmill and not cycle ergometers. There is a lack of information and relevant studies about the methodological aspects of the use of treadmill stress test and classic exercise protocols for the measurement of TWA. For example, the interference of physical exercise performed, or the noise generated in the TWA registers and the better way to confirm the obtained results.

Additionally, in this study we proposed to evaluate aspects of the TWA record in the 15 leads in three planes during the treadmill stress test, hypothesizing greater chances of detecting TWA at different points through this strategy, in contrast to what is currently recommended in the literature, for example, transverse plane and V5 lead analysis on exercise bicycle.

The lack of answers to these questions and the difficulties mentioned above were the incentives that motivated the accomplishment of the present study for basically a methodological evaluation. The basis of choosing the two types of exercise protocol on this trial (intense protocol like Ellestad and attenuated protocol such as Naughton) to evaluate the characteristics of the measurement and acquisition of TWA through the exercise test, was originated in pilot studies from our service which indicated a greater occurrence of noise, less validation and greater difficulty of measuring TWA values when intense exercise protocols were used.

The objective of study of this project is mainly an accurate and critical analysis at TWA acquisition methodology during exercise treadmill test. Larger updating factors such as 1/8 and 1/16 have been recommended, among the reasons is the fact that they are less subject to the influence of artifacts, ectopic beats and noise on the composition of the mean beat^26,27^. However, Verrier et al.^30^ recommend the factor 1/8 in the MMA analysis due to its high power to predict arrhythmic death, while the other factors (1/16, 1/32, and 1/64) should not be used for TWA analysis. Recently, Shah et al., on studying TWA and mental stress using the 1/32 update factor, suggests a correlation between increased TWA and mental stress. However, the values obtained were much lower than the 47 mV reference, probably due to the use of this factor^27^.

In our study, we found this same aspect of higher values of TWA regardless of the protocol or set of derivations analyzed for the groups with factor 1/8 in the different phases of the exam, as described by other authors in the literature^14,28^. In the present study, the performance of groups with factor 1/32 was considered poor when compared to 1/8, in terms of confirmation rate.

The phase of obtaining the highest value of TWA occurred in our study during the exercise phase in 88.1% of all exams, with a greater occurrence in Naughton groups than Ellestad groups. However, in the E8 group 18.1% of the tests presented the highest TWA during the recovery phase. This fact reiterates the attention to chest preparation before the exam and a comprehensive analysis of the entire exam and not just the exercise phase.

For TWA analysis it is recommended to consider only the values obtained in the sets of transverse plane and orthogonal leads, since the frontal plane would be affected by the influence of movement and noise, thus registering unrealistic values^28^. Our study did not show significantly different values between TWA measurements obtained in the frontal and transverse planes in the different groups studied, especially in the moment of registering the maximum TWA, with N8 group showing considerable confirmation not only in the transverse, but also in the 3 sets of leads. This allows a global and not limited analysis of TWA.

Noise found during the rest phase did not express any clinical importance. The smallest confirmation values for the N32 group may be associated to inadequate chest preparation and/or to a more physically limited patient profile.

The maximum TWA moment occurred predominantly during the exercise phase in this study, especially in the groups Naughton. The noise was significantly greater during the exercise phase, especially in the frontal plane set leads, and continued to be significant during recovery in the Ellestad groups. This could have been caused by a greater influence of the physical capacity, movement, breathing and muscular contraction associated to the Ellestad protocol.

In the isolated analysis of each set of leads the transverse plane showed a higher confirmation rate, especially in the N8 group. This was likely caused by the smaller influence of movement on the set of leads. On the other hand, the frontal leads had the worst performance during both exercise and recovery, probably caused by noise influence and movement interference. However, the Naughton groups showed good confirmation rates for the frontal plane.

In the analysis of noise values during the *exercise peak phase*, the Naughton groups showed about 50% confirmation rate for transverse and orthogonal sets of leads. The Ellestad groups, however, invalidated the measurements in practically 100% of the leads (heart rhythm > 125 bpm is always automatically disregarded by the software).

## Limitations

Limiting points of this study are its retrospective and non-randomized design, therefore the conditions of pre-test preparation and performance were not controlled by the researchers. The results of the analysis contemplated only 2 selected protocols, and their conclusions were limited to this universe.

## Conclusions

1. The study has the merit of clearly addressing the importance of methodological standardization for the improvement of TWA measurement during exercise testing.
2. The use of TWA analysis by MMA technique is feasible during the performance of an exercise treadmill test. We must considerer that, when using the Ellestad protocol, we need to consider performing TWA analysis restricted to the transverse plane.
3. The use of a protocol such as Naughton with an update factor of 1/8 allows a TWA analysis of the frontal, transverse and orthogonal leads with significant confirmation rates in each of these sets and in different phases of the treadmill test, including exercise peak.
4. The measurement of TWA incorporated in the clinical exercise test routine may contribute in an unequivocal way to the prognostic investigation of sudden cardiac death.

## Methodology

### Casuistry

A retrospective analysis of exercise tests that contained the TWA assessment tool was performed from a database of patients at a specialized tertiary cardiological unit, carried out between August 2006 and December 2014.

### Ethics

The Institutional Review Board of Hospital das Clinicas FMUSP, São Paulo, Brazil, approved this retrospective study (CAAE 67770417.4.0000.0068) by Approval # 2.128.88. Informed consent was waived by the Institutional Review Board since only the medical records of patients were retrospectively reviewed to obtain the necessary data for this research.

All methods were carried out in accordance with relevant guidelines and regulations for diagnostic methods.

### Inclusion criteria

Exercise tests data recorded in the medical records of individuals aged between 16 and 80 years were selected, through Naughton and Ellestad treadmill protocols and TWA updating factors 1/8 and 1/32. We choose the Ellestad protocol because it is applied to young, fit individuals, allowing an important increase in workloads at each stage; and the Naughton protocol for being considered attenuated with slight increments of load, generally applied to individuals with physical limitations and in cardiovascular rehabilitation.

### Exclusion criteria

Tests from medical records of individuals younger than 16 years or older than 80 years, performed using other types of exercise protocols or other updating factors, absence of TWA registration or retrieval were excluded.

### Analysis of exams

#### Exercise test performance: data from the included medical records

After adequate preparation of the chest, 14 electrodes were fixed for recording of 15 leads (12 classic ECG leads: frontal plane I, II, III, aVR, aVF, aVL; transverse plane V1 through V6; + 3 orthogonal leads X, Y, and Z). All tests were performed on a General Electric (GE, Milwaukee WI, USA,) treadmill, CASE 6.5 model, recording the rest, peak exertion, and recovery electrocardiograms. The interruption tests criteria followed the reference to intense physical fatigue (Borg scale 16 to 20) and classic interruption criteria for exercise treadmill.

#### Exercise protocols

In this study we used two of the protocols regularly employed in the routine of ergometry in our Institution, both modified in relation to their original publications. The Ellestad protocol is commonly applied for young individuals with some degree of physical activity, and is considered an example of an intense protocol due to its great workload increments at each step. The Naughton protocol, on the other hand, is applied for older, more physically limited individuals who follow cardiovascular rehabilitation programs, and is an example of an attenuated protocol, with little workload increments at each step. We display below a comparison between the two protocols.

**Table:** 

STEPS	Ellestad protocol modified			Naughton protocol modified		
	Time (min)	Velocity (mph)	Inclination (%)	Time (min)	Velocity (mph)	Inclination (%)
STEP 1	03	1.7	10.0	02	1.0	0.0
STEP 2	02	3.0	10.0	02	2.0	0.0
STEP 3	02	4.0	10.0	02	2.0	3.5
STEP 4	02	5.0	10.0	02	2.0	7.0
STEP 5	02	5.0	15.0	02	2.0	10.5
STEP 6	02	6.0	15.0	02	2.0	14.0
STEP 7	02	7.0	15.0	02	2.0	17.5
STEP 8	02	8.0	15.0	02	2.0	21.0

#### TWA measurement

**Figure 2:**
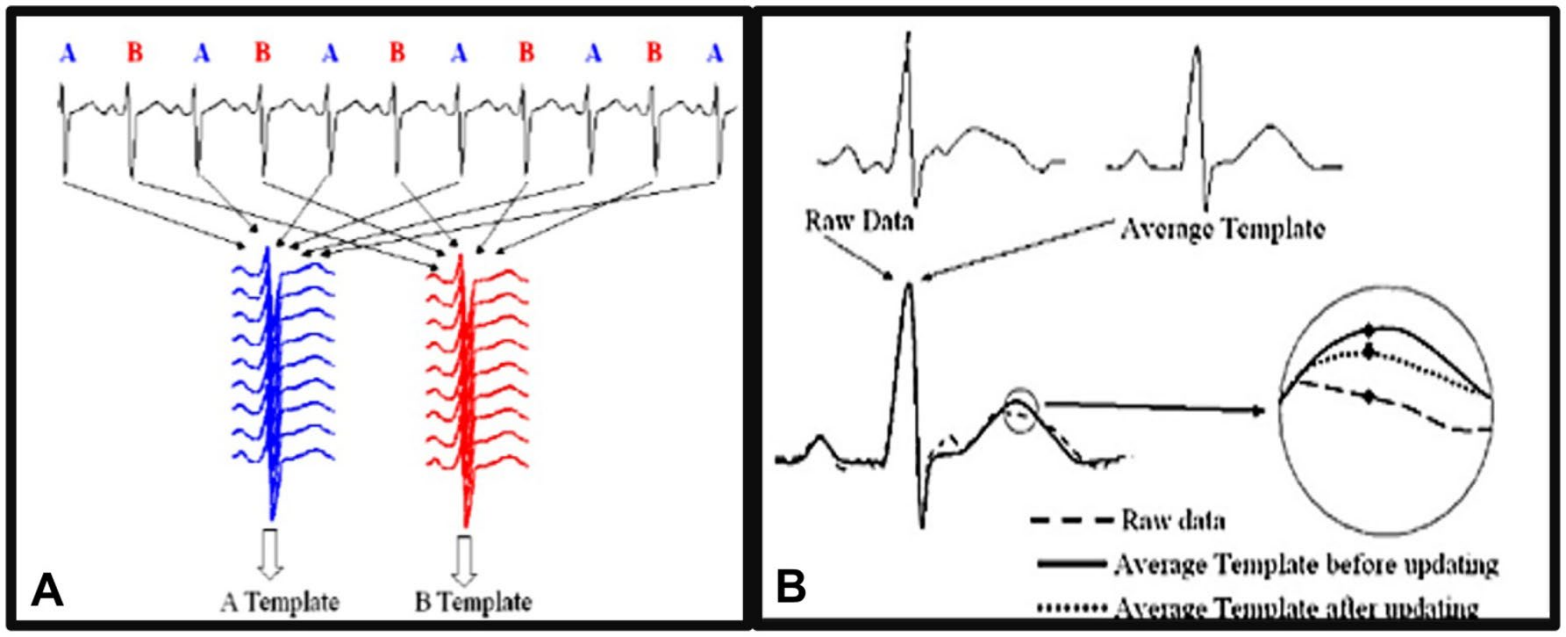
(**A**) Separation of beats into 2 categories A or B, even and odd, etc., successively during the performance of TWA by MMA method. (**B**) The model is updated by a small part of the difference between the old model and the new raw data (update factor or sensitivity, which can represent 1/8, 1/16, 1/32 or 1/64 of the difference between the mean and the new beat in question, and this value will be added to the average—“update”). Adapted from Verrier et al.^10,29^.

The TWA measurement obtained by MMA technique expresses the values of T-wave alternans up to heart rate of 125 bpm as the maximum difference in morphology between T waves for each period of 10 to 15 s in a sliding window^10–13,29^. There is a separation of the sequential beats between even (“A”) and odd (“B”) ones, as shown in Fig. 2A. Each new beat conducted is classified into one of two groups, and its contribution to the average is not integral, but only part of the difference between the current beat and the incoming beat is incorporated, this being defined by update factor^30^ 1/8, 1/16, 1/32 and 1/64 of the respective difference, as show in Fig. 2B. The TWA value obtained hypothetically approximates the “true” TWA final values, since it is an indirect measure of the alternance of myocardial action potentials.

**Figure 3:**
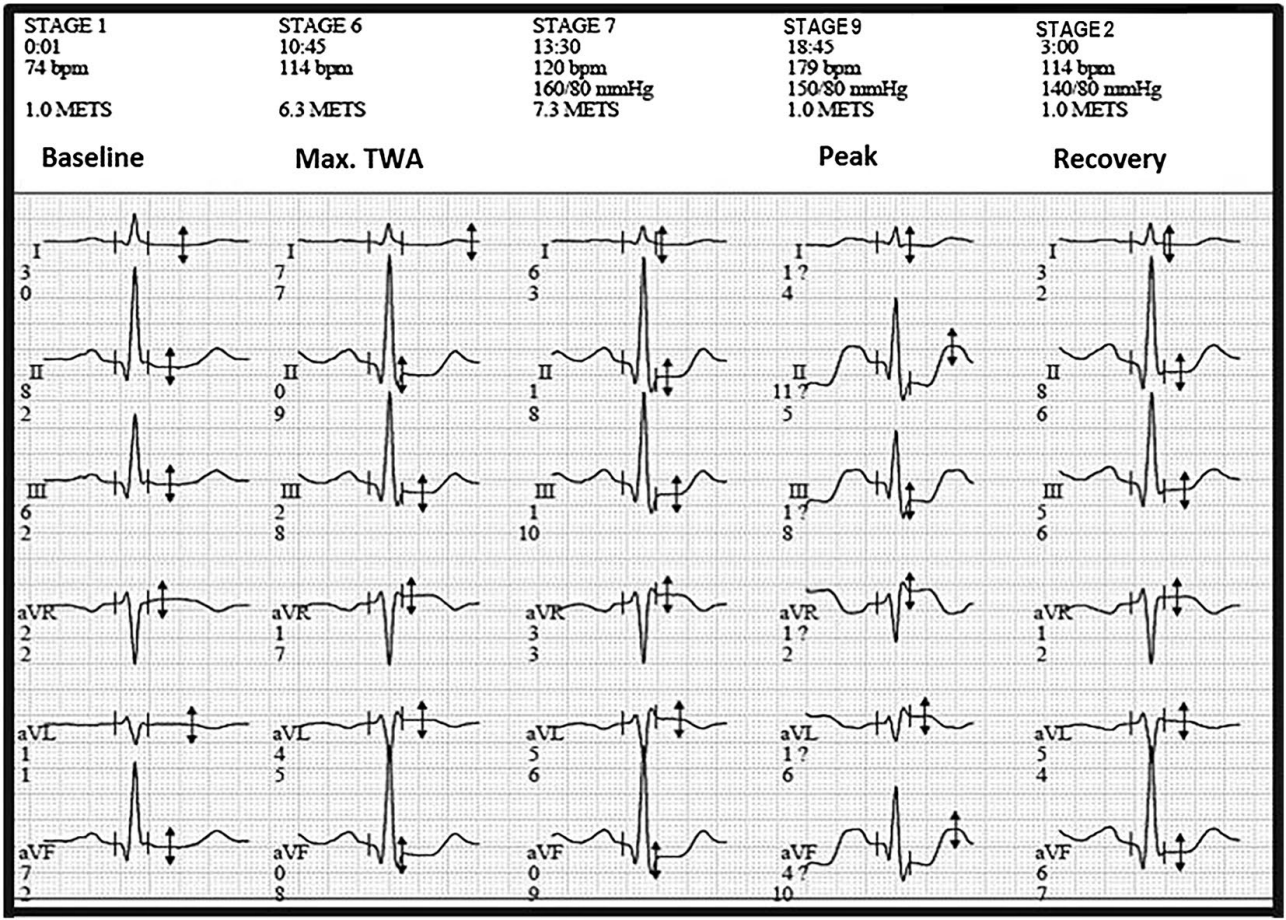
Aspect of the report generated at the end of the exam according to the leads and different moments of the exercise test, with the registration of TWA and noise with values expressed in microvolts (µV).

In addition to the software issuing a final report that expresses the TWA value and below this the noise value (Fig. 3), the results were confirmed by an independent examiner. When the noise level is above 20 microvolts, the TWA measures become doubtful and are expressed by the symbol “?”.

Even after the program has obtained the summary of TWA and noise measures, the examiners made a detailed review using the 15-s sliding windows, i.e., the software provides models of all the test phases every 15 s. This makes it possible to check if the values obtained by the program are trustful, reliable or not, and with that to carry out the acceptance or adjustment of the TWA values, allowing a visual confirmation of the results.

#### TWA confirmation

With 15 recorded leads, these were divided into 3 sets for confirmation analysis: frontal plane leads, transverse plane leads and orthogonal leads. Frontal and transverse planes must have at least 3 leads with valid measurements of TWA to be considered valid, and 2 leads of the orthogonal set.

#### Groups for analysis

After the selection, the exams were divided into 4 groups according to the exercise protocol (intense or attenuated) and the update factor: Ellestad protocol with 1/8 factor (E8), Ellestad protocol with 1/32 factor (E32), Naughton protocol with 1/8 factor (N8) and Naughton protocol with 1/32 factor (N32).

#### Variables studied

In this study we considered to evaluate the methodological question for the TWA value, noise value and confirmation by set of registered leads at the moments: rest, maximal TWA, recovery and peak of exercise.

### Statistical analysis

To study the normality of quantitative variables, the Kolmogorov–Smirnov test was applied. Student’s t test was used to compare the means of the two groups, and when the assumption of data normality was rejected the non-parametric Mann–Whitney test or Fisher’s exact test was used. In the significant results for the variables TWA, noise and Validation, a two-by-two comparison was performed to identify which groups had a significant difference. In this moment, the Mann–Whitney tests were used for numerical variables, and Fisher’s exact test for the categorical variables, considering the Bonferroni correction. The significance level considered for the tests was 5%. The analyses were performed with the statistical software R (R core Team 2021).

## Data Availability

Materials described in the manuscript, including all relevant raw data, will be freely available to any researcher wishing to use them for non-commercial purposes, without breaching participant confidentiality, upon request to the authors. Please contact the author Dr. Horacio Gomes Pereira Filho, MD, PhD, at hgpfilho@gmail.com.
